# Epidemic Cholera in a Crowded Urban Environment, Port-au-Prince, Haiti

**DOI:** 10.3201/eid1711.110772

**Published:** 2011-11

**Authors:** Stacie E. Dunkle, Adamma Mba-Jonas, Anagha Loharikar, Bernadette Fouché, Mireille Peck, Tracy Ayers, W. Roodly Archer, Valery M. Beau De Rochars, Thomas Bender, Daphne B. Moffett, Jordan W. Tappero, George Dahourou, Thierry H. Roels, Robert Quick

**Affiliations:** Centers for Disease Control and Prevention, Atlanta, Georgia, USA (S.E. Dunkle, A. Mba-Jonas, A. Loharikar, T. Ayers, W.R. Archer, V.M. Beau De Rochars, T. Bender, D.B. Moffett, J.W. Tappero, G. Dahourou, T.H. Roels, R. Quick); Ministry of Public Health and Population, Port-au-Prince, Haiti (B. Fouché); Haitian Group for the Study of Kaposi’s Sarcoma and Opportunistic Infections, Port-au-Prince (M. Peck)

**Keywords:** bacteria, epidemic, waterborne infections, cholera, enteric infections, Vibrio cholerae, case–control studies, Haiti, urban population, hygiene, handwashing, diet, prevention and control, dispatch

## Abstract

We conducted a case–control study to investigate factors associated with epidemic cholera. Water treatment and handwashing may have been protective, highlighting the need for personal hygiene for cholera prevention in contaminated urban environments. We also found a diverse diet, a possible proxy for improved nutrition, was protective against cholera.

Epidemic cholera remains a problem in poor countries that lack adequate water and sanitation infrastructure, particularly among populations in crowded, unsanitary conditions (*1**–**4*). On January 12, 2010, a magnitude 7.0 earthquake struck metropolitan Port-au-Prince, Haiti, killing >200,000 persons and destroying vital water and sanitation infrastructure (*5*). Epidemic cholera had not been reported in Haiti in the past century, but on October 21, 2010, toxigenic *Vibrio cholerae* serogroup O1, serotype Ogawa, biotype El Tor, was identified as the cause of a large outbreak of acute watery diarrhea in Artibonite Department, ≈150 km north of Port-au-Prince (*6*). By November 7, the outbreak had reached Port-au-Prince, where >1 million persons were living in internally displaced person camps or crowded slums. By December 15, ≈20,000 cases had been reported in the capital (*7*). We conducted a case–control study during December 15–19, 2010, to investigate illness transmission and guide public health actions.

## The Study

We defined a case as acute, watery diarrhea in a person >5 years of age admitted to the Haitian Group for the Study of Kaposi’s Sarcoma and Opportunistic Infections (GHESKIO) cholera treatment center (CTC) in Cité de Dieu slum after November 1, 2010. Enumerators administered a standard questionnaire in Haitian Creole to CTC patients or their caregivers to gather demographic, clinical, and treatment information; food and beverage exposures in the 3 days before illness onset; and water, sanitation, and hygiene practices. Enumerators visited each case-patient’s household to observe living conditions; water storage and treatment practices; and handwashing technique, which included an assessment of soap use, lathering, and drying procedure. Enumerators enrolled 2 sex-, age group– (5–15 years, 16–30 years, 31–45 years, and >45 years), and neighborhood-matched controls per case-patient by skipping the immediate neighbor and going house to house until 2 controls were identified. An identical questionnaire that included household observations was administered to controls.

We used exact conditional logistic regression to compute matched odds ratios (mORs) with 95% confidence intervals (CIs). For protective food exposures, we calculated a food diversity score for each participant based on the total number of distinct food items consumed in the 3 days before illness onset. We created a 2-level categorical diversity score variable based on the median score. The study protocol was approved by the Haitian Ministry of Public Health and Prevention and the GHESKIO institutional review board.

We enrolled 53 case-patients and 106 controls. The median ages of case-patients and controls were 29 (range 6–80) and 30 (range 6–85) years, respectively; 45% of case-patients and controls were female. Of participants >15 years of age, 84% self-reported as literate; 37% of case-patients and 57% of controls spoke French (mOR 0.3, 95% CI 0.1–0.8). Participant households were located in the greater Port-au-Prince area (Figure).

**Figure:**
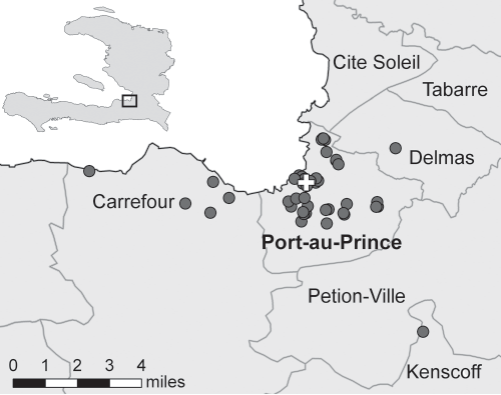
Locations of the Haitian Group for the Study of Kaposi’s Sarcoma and Opportunistic Infections Cholera Treatment Center and case-patient households in Port-au-Prince, Haiti, 2010. Cross indicates cholera treatment center location; circles indicate households.

All case-patients reported having acute, watery diarrhea; other signs and symptoms included vomiting (81%), rice-water stool (66%), and leg cramps (64%). Reported illness onset dates ranged from December 10 through 19. Of 53 case-patients, 20 (38%) were treated with oral rehydration solution at home prior to admission, and 39 (74%) sought care at a GHESKIO CTC on the first day of symptom onset. CTC treatment included ORS (85%), intravenous fluids (55%), and antimicrobial drugs (9%).

Water sources, which included purchased bags, purchased bottled or filtered water, piped water, and water collected from a tanker, did not differ between case-patients and controls (Table 1). Bladder water (chlorinated water stored in flexible plastic tanks in internally displaced person camps) seemed protective (mOR indeterminate, 95% CI 0–0.9), although exposure frequency was limited and a point estimate could not be calculated. Controls were more likely than case-patients to have treated their drinking water by boiling or chlorinating before the outbreak began in Port-au-Prince (mOR 0.3, 95% CI 0.1–0.9) and to have used proper handwashing technique (mOR 0.2, 95% CI 0.03–0.7).

**Table 1:** Characteristics of cholera case-patients and controls, Port-au-Prince, Haiti, December 2010*

Variable	No. (%) case-patients, n = 53	No. (%) controls, n = 106	mOR (95% CI)
Socioeconomic			
Completed primary school†	5 (29)‡	13 (36)‡	0.2 (0.0–1.7)
Literate†	37 (84)‡	72 (84)‡	1.0 (0.3–3.9)
French speaking†	16 (36)‡	49 (57)‡	0.3 (0.1–0.8)
Has electricity	29 (55)	71 (67)‡	0.5 (0.2–1.2)
Owns a radio	37 (70)	77 (73)	0.8 (0.3–2.2)
Owns a television	26 (50)‡	58 (55)‡	0.8 (0.4–1.8)
Owns a car/motorcycle	3 (6)‡	12 (12)‡	0.4 (0.1–1.9)
IDP camp (self-reported)	15 (28)	24 (23)	2.1 (0.5–8.7)
IDP camp (observed)	10 (24)‡	19 (24)‡	0.7 (0.1–7.3)
Tarp roof	11 (21)	19 (18)	1.6 (0.3–8.8)
Unemployed§	5 (13)‡	14 (18)‡	0.7 (0.2–2.4)
Water sources			
Purchased bags (sachets)	9 (17)	9 (9)	2.6 (0.7–10.2)
Purchased bottles/filter	12 (23)	25 (24)	0.9 (0.4–2.3)
Piped (house, yard, public tap)	25 (47)	58 (55)	0.6 (0.2–1.6)
Tanker	7 (13)	11 (10)	1.4 (0.4–5.0)
Bladder	0	8 (8)	NA (0.0–0.9)
Water treatment and handwashing			
Boiling water or using a chlorine product <3 d before illness	37 (70)	86 (81)	0.5 (0.2–1.2)
Boiling water or using a chlorine product before November 1, 2010	37 (70)	90 (85)‡	0.3 (0.1–0.9)
Proper handwashing	8 (15)	31 (29)	0.2 (0.0–0.7)
Sanitation: access to toilet/latrine			
	45 (85)‡	97 (92)‡	0.5 (0.1–1.7)
Foods			
Food or drink from a street vendor	23 (47)‡	52 (55)‡	0.7 (0.3–1.7)
Cold leftover food	23 (44)‡	59 (56)	0.6 (0.2–1.3)
Cold rice	30 (58)‡	59 (56)‡	1.1 (0.5–2.5)
Raw food	3 (6)‡	5 (5)‡	1.2 (0.2–6.2)
Seafood	12 (23)	37 (35)	0.5 (0.2–1.2)
Food diversity (>23 items)	15 (28)	59 (56)	0.3 (0.1–0.6)

*mOR, matched odds ratio; CI, confidence interval; IDP, internally displaced person. †Respondents >15 y of age. ‡Denominator does not include all respondents. §Respondents >17 y of age.

Food exposures implicated as risk factors for transmission in previous cholera outbreaks in the Americas were not associated with illness, including food or drink purchased from a street vendor, cold leftover food, cold rice, raw food, and seafood (Table 1). Of 60 food exposures included in the questionnaire for the 3 days before illness onset, 29 (48%) were protective against cholera; CIs did not overlap. The median food diversity score for case-patients and controls in the 3 days before illness was 23 (range 4–50). A higher percentage of controls (56%) than case-patients (28%) consumed more than the median number of 23 items in the 3 days before illness (mOR 0.3, 95% CI 0.1–0.6).

Food diversity score, proper handwashing, and treating drinking water were included in a multivariate model (Table 2). All 3 remained protective against illness, although treating drinking water did not reach statistical significance (mOR 0.4, 95% CI 0.1–1.1). All socioeconomic status variables were considered for model inclusion, but none affected the direction or effect size of predictor variables for cholera.

**Table 2:** Practices independently associated with cholera prevention in a multivariate model case–control study, Port-au-Prince, Haiti, December 2010*

Practice	mOR (95% CI)	p value
Food diversity (>23 items)	0.3 (0.1–0.7)	<0.01
Proper handwashing	0.2 (0.03–0.90)	0.03
Boiling water or using a chlorine product	0.4 (0.1–1.1)	0.08

*mOR, matched odds ratio; CI, confidence interval.

## Conclusions

In this investigation, we identified 2 key practices that may have protected against cholera in the contaminated urban environment of Port-au-Prince: habitual water treatment and proper handwashing. These findings were biologically plausible and consistent with the findings of cholera investigations in other settings (*3**,**8**–**11*).

The protective effect of numerous food exposures was difficult to interpret, although food items such as rice, dried fish, and citrus fruit juice have been found to decrease the risk for cholera in previous outbreaks (*8**,**11**,**12*). We explored the role of food diversity through the calculated score, summarizing the relationship with illness by using crude categorization based on the median number of food items consumed, and found that food diversity was protective against illness. This finding was similar to the protective effect of diet variability observed in a case–control study of illness caused by *Escherichia coli* O157:H7 (*13*). Although differences in food diversity may serve as a proxy for socioeconomic status, other socioeconomic status variables included in multivariate models did not adjust for the protective effect of handwashing and treating water. Alternatively, food diversity differences between case-patients and controls may be a result of differential reporting by case-patients during disease incubation or may reflect the nutritional benefit of a more varied diet, which may mitigate the risk for illness. Further research into the role of diet diversity in diarrheal disease outbreaks is warranted.

This investigation revealed no risk factors for illness despite the inclusion in the questionnaire of numerous potential food and drink exposures identified in hypothesis-generating interviews, including several previously implicated in cholera outbreaks (*14*). The rapid, explosive spread of cholera across Haiti and within Port-au-Prince made it unlikely that a point source would be identified. Instead, poor sanitary infrastructure and widespread contamination created ideal conditions for propagated disease dissemination through multiple vehicles (*15*). In such circumstances, the attributable risk for individual exposures may decrease while personal protective measures, such as household water treatment and handwashing with soap, may emerge as noteworthy findings.

After the January 2010 earthquake, the population of Port-au-Prince was vulnerable to disease outbreaks because of problems with overcrowding, poverty, poor nutrition, and inadequate water and sanitation infrastructure. The cholera epidemic, which was unexpected and particularly explosive in this immunologically naive population, strained the country’s capacity to respond. Personal hygiene measures taken by persons and families were crucial to protect against disease. In the long term, with cholera likely to remain a problem in Haiti, providing water and sanitation infrastructure should be a high priority for government and aid organizations.

## References

[1] Ries AA, Vugia DJ, Beingolea L, Palacios AM, Vasquez E, Wells JG, Cholera in Piura, Peru: a modern urban epidemic. J Infect Dis. 1992;166:1429–33. 10.1093/infdis/166.6.1429 1431259

[2] Swerdlow DL, Mintz ED, Rodriguez M, Tejada E, Ocampo C, Espejo L, Waterborne transmission of epidemic cholera in Trujillo, Peru: lessons for a continent at risk. Lancet. 1992;340:28–33. 10.1016/0140-6736(92)92432-F 1351608

[3] Weber JT, Mintz ED, Canizares R, Semiglia A, Gomez I, Sempertegui R, Epidemic cholera in Ecuador: multidrug-resistance and transmission by water and seafood. Epidemiol Infect. 1994;112:1–11. 10.1017/S0950268800057368 8119348 PMC2271476

[4] Swerdlow DL, Malenga G, Begkoyian G, Nyangulu D, Toole M, Waldman RJ, Epidemic cholera among refugees in Malawi, Africa: treatment and transmission. Epidemiol Infect. 1997;118:207–14. 10.1017/S0950268896007352 9207730 PMC2808810

[5] Centers for Disease Control and Prevention. Post-earthquake injuries treated at a field hospital—Haiti, 2010. MMWR Morb Mortal Wkly Rep. 2011;59:1673–7. 21209607

[6] Centers for Disease Control and Prevention. Cholera outbreak—Haiti, October 2010. MMWR Morb Mortal Wkly Rep. 2010;59:1411. 21048563

[7] Republic of Haiti Ministry of Public Health and Population. 2010 Dec 15. [cited 2011 Aug 18]. http://www.mspp.gouv.ht/site/index.php?option=com_content&view=article&id=57&Itemid=1

[8] DuBois AE, Sinkala M, Kalluri P, Makasa-Chikoya M, Quick RE. Epidemic cholera in urban Zambia: hand soap and dried fish as protective factors. Epidemiol Infect. 2006;134:1226–30. 10.1017/S0950268806006273 16623992 PMC2870514

[9] Reller ME, Mong YJ, Hoekstra RM, Quick RE. Cholera prevention with traditional and novel water treatment methods: an outbreak investigation in Fort-Dauphin, Madagascar. Am J Public Health. 2001;91:1608–10. 10.2105/AJPH.91.10.1608 11574318 PMC1446837

[10] St Louis ME, Porter JD, Helal A, Drame K, Hargrett-Bean N, Wells JG, Epidemic cholera in West Africa: the role of food handling and high-risk foods. Am J Epidemiol. 1990;131:719–28. 2316500 10.1093/oxfordjournals.aje.a115556

[11] Quick RE, Thompson BL, Zuniga A, Dominguez G, De Brizuela EL, De Palma O, Epidemic cholera in rural El Salvador: risk factors in a region covered by a cholera prevention campaign. Epidemiol Infect. 1995;114:249–55. 10.1017/S0950268800057915 7705488 PMC2271272

[12] Mujica OJ, Quick RE, Palacios AM, Beingolea L, Vargas R, Moreno D, Epidemic cholera in the Amazon: the role of produce in disease risk and prevention. J Infect Dis. 1994;169:1381–4. 10.1093/infdis/169.6.1381 8195622

[13] Kassenborg HD, Hedberg CW, Hoekstra M, Evans MC, Chin AE, Marcus R, Farm visits and undercooked hamburgers as major risk factors for sporadic *Escherichia coli* O157:H7 infection: data from a case–control study in 5 FoodNet sites. Clin Infect Dis. 2004;38(Suppl 3):S271–8. 10.1086/381596 15095199

[14] Tauxe RV, Mintz ED, Quick RE. Epidemic cholera in the new world: translating field epidemiology into new prevention strategies. Emerg Infect Dis. 1995;1:141–6. 10.3201/eid0104.950408 8903186 PMC2626892

[15] Hill VH, Cohen NJ, Kahler AM, Jones JL, Bopp CA, Marano N, Detection of toxigenic *Vibrio cholerae* O1 in water and seafood samples, Haiti. Emerg Infect Dis. 2011;17:2147–50. 10.3201/eid1711.110748 PMC3310574 22099121

